# Nasal Obstructions and Spinal Deformities

**Published:** 1891-03

**Authors:** 


### Nasal Obstructions and Spinal Deformities.

While investigating the rachitic deformities of the spine, Dr. Redard was struck by the fact that these were frequently found in persons suffering from nasal obstruction. On the ground of his findings he considers himself warranted in formulating the following conclusions:

1. Nasal occlusion is a frequent cause of kyphoscoliosis, or deformity of the chest.

2. Scoliosis due to this cause is usually dorsal and slightly marked, but attended with important deformities of the chest, especially in females, and is developed during the period of growth in consequence of protracted inflammation of the mucous membrane of the upper air passages.

3. The nasal obstruction is mainly due to adenoid vegetations.

4. The treatment of the nasal trouble causes rapid improvement of certain forms of kyphosis, scoliosis, and abnormalities of the thorax.—*Gazette Medical de Paris*, No. 12, 1890.

